# Male breast cancer in a multi-gene panel testing cohort: insights and unexpected results

**DOI:** 10.1007/s10549-016-4085-4

**Published:** 2016-12-22

**Authors:** Mary Pritzlaff, Pia Summerour, Rachel McFarland, Shuwei Li, Patrick Reineke, Jill S. Dolinsky, David E. Goldgar, Hermela Shimelis, Fergus J. Couch, Elizabeth C. Chao, Holly LaDuca

**Affiliations:** 1 Ambry Genetics, Aliso Viejo, CA USA; 2 Department of Dermatology, Huntsman Cancer Institute, University of Utah, Salt Lake City, UT USA; 3 Department of Laboratory Medicine and Pathology, Mayo Clinic, Rochester, MN USA; 4 Department of Health Sciences Research, Mayo Clinic, Rochester, MN USA; 5 Division of Genetics and Genomics, Department of Pediatrics, University of California, Irvine, Irvine, CA USA

**Keywords:** Male breast cancer, Multi-gene panel testing, *BRCA2*, *CHEK2*, *PALB2*

## Abstract

**Purpose:**

Genetic predisposition to male breast cancer (MBC) is not well understood. The aim of this study was to better define the predisposition genes contributing to MBC and the utility of germline multi-gene panel testing (MGPT) for explaining the etiology of MBCs.

**Methods:**

Clinical histories and molecular results were retrospectively reviewed for 715 MBC patients who underwent MGPT from March 2012 to June 2016.

**Results:**

The detection rate of MGPT was 18.1% for patients tested for variants in 16 breast cancer susceptibility genes and with no prior *BRCA1/2* testing. *BRCA2* and *CHEK2* were the most frequently mutated genes (11.0 and 4.1% of patients with no prior *BRCA1/2* testing, respectively). Pathogenic variants in *BRCA2* [odds ratio (OR) = 13.9; *p* = 1.92 × 10^−16^], *CHEK2* (OR = 3.7; *p* = 6.24 × 10^−24^), and *PALB2* (OR = 6.6, *p* = 0.01) were associated with significantly increased risks of MBC. The average age at diagnosis of MBC was similar for patients with (64 years) and without (62 years) pathogenic variants. *CHEK2* 1100delC carriers had a significantly lower average age of diagnosis (*n* = 7; 54 years) than all others with pathogenic variants (*p* = 0.03). No significant differences were observed between history of additional primary cancers (non-breast) and family history of male breast cancer for patients with and without pathogenic variants. However, patients with pathogenic variants in *BRCA2* were more likely to have a history of multiple primary breast cancers.

**Conclusion:**

These data suggest that all MBC patients regardless of age of diagnosis, history of multiple primary cancers, or family history of MBC should be offered MGPT.

**Electronic supplementary material:**

The online version of this article (doi:10.1007/s10549-016-4085-4) contains supplementary material, which is available to authorized users.

## Introduction

While the incidence of male breast cancer (MBC) in the general population is low (1:1000), it can be significantly elevated for patients with an underlying genetic predisposition. Comprehensive genetics evaluation of all MBC patients is important, as identification of various cancer-predisposing mutations can drastically impact medical management for patients and their family members. The *BRCA1* and *BRCA2* genes, implicated in hereditary breast and ovarian cancer syndrome (HBOC), have been associated with increased risks for MBC, and it is currently recommended that individuals with a personal or family history of male breast cancer undergo testing of these genes [1]. *BRCA2* is the most frequently mutated gene in MBC cohorts, having been reported in 4–40% of MBC patients, depending on the population studied and the presence/absence of additional clinical history supporting a diagnosis of HBOC [2–9]. Cumulative lifetime breast cancer risks for male *BRCA1* and *BRCA2* pathogenic variant carriers are 1–2 and 5–10%, respectively. In addition to breast cancer, males with *BRCA1* or *BRCA2* pathogenic variants face increased lifetime risks for prostate and pancreatic cancers [10–12].

Beyond *BRCA1/BRCA2*, data are limited regarding genetic predisposition to MBC. Two independent studies have linked *CHEK2* 1100delC with MBC [13, 14]; however, results from multiple other studies have not confirmed this association [15–22]. Furthermore, the role of other *CHEK2* pathogenic variants in MBC is yet to be explored. Germline pathogenic variants in the *PTEN*, androgen receptor (*AR*), *NF1*, and *PALB2* genes have also been reported in MBC patients; however, associations with MBC have not been well-studied and risk estimates are not currently available [23–26].

The clinical availability of multi-gene panel testing (MGPT) presents an opportunity for patients to undergo comprehensive analysis of a wide range of cancer susceptibility genes, including those with and without established links to MBC. Despite increased utilization of such testing in hereditary cancer diagnostics, data remain limited regarding the yield of such testing for MBC patients. In a recent study of breast cancer patients who underwent MGPT, 31.8% (*n* = 7/22) of MBC cases tested positive for pathogenic or likely pathogenic variants: *BRCA1* (1)*, BRCA2* (3), *PALB2* (1), *CHEK2* (1), and *ATM* (1) [8]. These results are yet to be validated in larger MBC cohorts. To better understand the genetic contribution to MBC and the yield of MGPT in this population, we retrospectively assessed a cohort of MBC patients referred for MGPT.

## Methods

### Study population

Clinical histories and molecular results were retrospectively reviewed for all MBC patients (*n* = 715) who underwent MGPT at Ambry Genetics between March 2012 and June 2016 (Aliso Viejo, CA). The following demographic and clinical history information was obtained from test requisition forms and clinic notes submitted by ordering providers: age at testing, ethnicity, *BRCA1/2* testing history, and personal/family cancer history. Patients were excluded if they were known *BRCA1/2* pathogenic variant carriers prior to MGPT (*n* = 1), if heterozygosity ratios of less than <25% were observed for any reported alterations detected in the patient (*n* = 3), or if the only information suggesting a MBC diagnosis was an ICD-9 code (*n* = 3), leaving 708 MBC patients eligible for further study.

### Laboratory methods

Patients underwent comprehensive analysis of cancer susceptibility genes using a variety of gene panels (Online Resource 1). Genomic deoxyribonucleic acid (gDNA) was isolated from the patient’s blood or saliva specimen using a standardized methodology (Qiagen, Valencia, CA) and quantified by spectrophotometer (Nanodrop; Thermoscientific, Pittsburgh, PA, or Infinite F200; Tecan, San Jose, CA). Sequence enrichment was performed by incorporating the gDNA onto a microfluidics chip or into microdroplets along with primer pairs or by a bait-capture methodology using long biotinylated oligonucleotide probes (Fluidigm, South San Francisco, CA, RainDance Technologies, Billerica, MA or Integrated DNA Technologies, San Diego, CA), followed by PCR and NGS analysis (Illumina, San Diego, CA) of all coding regions ± five bases into introns and untranslated regions (5′UTR and 3′UTR). Sanger sequencing was performed for any regions with insufficient depth of coverage for reliable heterozygous variant detection and for verification of variant calls, other than known non-pathogenic alterations. A targeted chromosomal microarray was used for the detection of gross deletions and duplications for each sample (Aglient, Santa Clara, CA). Initial data processing and base calling were performed with RTA 1.12.4 (HiSeq Control Software 1.4.5; Illumina). Sequence quality filtering was executed with CASAVA software (version 1.8.2; Illumina, Hayward, CA). Sequence fragments were aligned to the reference human genome (GRCh37), and variant calls were generated using CASAVA. A minimum quality threshold of Q20 was applied, translating to an accuracy of >99.9% for the called bases.

### Variant classification

Variants were annotated with the Ambry Variant Analyzer, a proprietary alignment and variant annotation software (Ambry Genetics) that assigned variants according to a five-tier variant classification protocol [pathogenic mutation; variant, likely pathogenic (VLP); variant of unknown significance (VUS); variant, likely benign (VLB); and benign], based on published recommendations from the American College of Medical Genetics and Genomics and the International Agency for Research on Cancer [27–29].

### Statistical analysis

The frequency of pathogenic or likely pathogenic variants was calculated for *ATM*, *BARD1*, *BRCA1*, *BRCA2*, *BRIP1*, *CDH1*, *CHEK2*, *MRE11A*, *NBN*, *NF1*, *PALB2*, *PTEN*, *RAD50*, *RAD51C*, *RAD51D*, and *TP53*. To avoid potential bias introduced by prior *BRCA1/2* testing and the varying number of genes tested by panel type, the diagnostic yield of MGPT was assessed using MBC patients tested for all 16 breast cancer genes (*n* = 512) and then stratified by prior *BRCA1/2* testing status. Clinical history comparisons were performed using patients tested for all 16 breast cancer genes, after removal of cases with pathogenic variants in genes not associated with breast cancer (*n* = 6), multiple pathogenic variants in breast cancer genes (*n* = 5), patients with monoallelic *MUTYH* pathogenic variants as the only pathogenic variant detected (*n* = 5), and patients carrying the low-risk *CHEK2* p.I157T variant (*n* = 6). Multivariable logistic regression (controlling for age, ethnicity and panel ordered) was performed to compare personal history of additional primary cancers and family history of MBC. A two-sample *t* test was used to test the age difference between groups.

### Breast cancer risk estimation

Among 708 MBC patients, 538 were Caucasian or Ashkenazi Jewish. Of these, individuals tested for all 16 breast cancer predisposition genes (*n* = 421) were subjected to breast cancer risk estimation. The non-Finn European population (NFE) in the Exome Aggregation Consortium (ExAC) dataset [30], excluding The Cancer Genome Atlas (TCGA) exomes, were used as public controls for case–control association studies with Caucasian breast cancer cases, consistent with the effective use of this dataset for estimation of ovarian and prostate cancer risk in recent studies [31, 32]. ExAC filter PASS/non-PASS rather than PASS only variants from the ExAC NFE-non TCGA dataset were used because multiple pathogenic variants validated by Ambry Genetics were excluded from the filter PASS category of ExAC. Restricting to PASS only variants led to reduced numbers of variants in controls and inflated breast cancer risks associated with each gene. To account for low-quality ExAC variants, recurrent variants observed at significantly different frequencies in other populations or with sequence misalignment were excluded. All remaining loss of function variants (nonsense, frameshift, consensus dinucleotide splice site (±1 or 2), and any missense variants defined as pathogenic in ClinVar by clinical laboratories) in breast cancer cases and ExAC controls were selected for inclusion. A series of filtering steps were applied (Supplementary Methods) to normalize differences in the breast cancer cases and the ExAC controls. Breast cancer cases carrying two or more pathogenic variants were excluded because of potential for inflation of breast cancer risks. While this filter was not applied to ExAC data due to the absence of individual-level genotype data, these events are rare in the general population and should only have a minor, conservative impact on risks estimates. Similarly, large genomic rearrangements of one or more exons were excluded from cases and ExAC controls because rearrangements were not validated among controls. Sensitivity analyses were also conducted when restricting to cases without prior *BRCA1/2* testing, to account for ascertainment bias (*n* = 268). Associations with breast cancer were estimated using the Fisher’s exact test.

## Results

### Demographics

This cohort was primarily Caucasian (66.1%), with other ethnicities each representing ≤10% of patients tested (Table 1). Ethnicity was unspecified for 6.2% of the cohort. The majority of patients were aged 60 and older at the time of testing (71.7%) and at the time of first breast cancer diagnosis (61.0%). Four percent of MBC patients had a second primary breast cancer, and additional non-breast primary cancers were reported for 23.4%. The most common additional cancer was prostate cancer, which was significantly enriched in this cohort with a frequency of 9.5% (*n* = 67) compared with the general population (0.13%; *p* = 10^−16^) [33]. A family history of MBC was reported for 6.4% of patients.

**Table 1:** Demographics of overall male breast cancer cohort (*n* = 708)

Demographic	*N*	Total	%
Ethnicity			
Caucasian	468	708	66.1
Ashkenazi Jewish	70	708	9.9
African American	58	708	8.2
Asian	23	708	3.2
Hispanic	16	708	2.3
Middle Eastern	5	708	0.7
Native American	1	708	0.1
Mixed ethnicity	20	708	2.8
Other	3	708	0.4
Unknown	44	708	6.2
Panel ordered (total number of genes on panel)			
BRCAplus (5–6)	115	708	16.2
GYNplus (9–13)	7	708	1.0
BRCAplus-expanded	17	708	2.4
BreastNext (14–18)	297	708	41.9
OvaNext (19–24)	66	708	9.3
PancNext (range)	5	708	0.7
CancerNext (22–32)	148	708	20.9
CancerNext-expanded (43–49)	53	708	7.5
Age at testing			
20–29	2	708	0.3
30–39	18	708	2.5
40–49	43	708	6.1
50–59	138	708	19.5
60–69	234	708	33.1
70–79	189	708	26.7
80–89	75	708	10.6
90 and older	9	708	1.3
Age at diagnosis^a^			
20–29	10	687	1.5
30–39	28	687	4.1
40–49	70	687	10.2
50–59	160	687	23.3
60–69	210	687	30.6
70–79	158	687	23.0
80–89	47	687	6.8
90 and older	4	687	0.6
Testing history			
Prior *BRCA1/2* testing	223	708	31.5
Clinical History^a^			
Family history male breast cancer	41	643	6.4
Multiple primary breast cancers	28	706	4.0
Additional non-breast primary cancers	166	708	23.4

^a^Age at diagnosis and clinical history were not provided for all men in the cohort

### Test results

Ninety-seven of 708 MBC patients were found to have at least one pathogenic or likely pathogenic variant in a breast cancer susceptibility gene (Table 2). Seven of these patients were found to carry two pathogenic variants including one biallelic *ATM* carrier with a clinical diagnosis of ataxia-telangiectasia, two *ATM/BRCA2* carriers, one *BRIP1/BRCA2* carrier, one *BRCA1/CHEK2* carrier, one *BARD1/PALB2* carrier, and one *CHEK2/PALB2* carrier. *BRCA2* and *CHEK2* were the most frequently altered genes, with pathogenic variants identified in 11.0 and 4.1% of MBC patients with no prior *BRCA1/2* testing, respectively (Table 3). No pathogenic variants were identified in the following hereditary breast cancer genes: *CDH1*, *PTEN*, *RAD50*, *RAD51C*, and *TP53*.

**Table 2:** Clinical histories of mutation carriers (N = 97)

Positive gene(s)	Pathogenic variant(s)	First breast cancer age	Bilateral/multiple breast cancers	Other cancer(s)	Family history of MBC^a^	Ethnicity	Notes
*ATM*	p.K2756*	70–79	No	Liver/melanoma/prostate	NP	Caucasian	
*ATM*	p.R2832C	60–69	No		No	Caucasian	
*ATM*	c.901+1G>A	70–79	No		No	Hispanic	
*ATM/ATM*	c.5763-1050A>G/c.8418+5_8418+8delGTGA	50–59	No		No	Caucasian	AT clinical dx
*ATM/BRCA2*	p.W2638*/c.5616_5620delAGTAA	70–79	No	Prostate	No	African American	
*ATM/BRCA2*	p.Q2651*/p.Q548*	80–89	No	Skin	No	Caucasian	
*BARD1*	p.Q564*	70–79	No		NP	Caucasian	
*BARD1/PALB2*	c.1935_1954dup20/c.109-2A>G	50–59	No		Yes	Unknown	
*BRCA1*	EX11_13del	50–59	No		No	Caucasian	
*BRCA1*	p.C61G	60–69	No		No	Caucasian	
*BRCA1*	EX11_13del	60–69	No		No	Caucasian	
*BRCA1*	c.5266dupC	60–69	No		No	Caucasian	
*BRCA1*	c.3481_3491del11	60–69	No	Liver	No	Caucasian	
*BRCA1/CHEK2*	c.5177_5180delGAAA/c.1100delC	40–49	No		No	African American	
*BRCA2*	5′UTR_EX1del	50–59	No	Bladder	NP	Caucasian	
*BRCA2*	c.5576_5579delTTAA	60–69	No		NP	Caucasian	
*BRCA2*	c.9253dupA	60–69	No		NP	African American	
*BRCA2*	p.R2520*	60–69	Yes		NP	Middle Eastern	
*BRCA2*	c.1813dupA	70–79	No	Prostate	NP	Caucasian	
*BRCA2*	5′UTR_EX1del	60–69	No	Gastroesophageal	No	Caucasian	
*BRCA2*	c.518delG	60–69	No	Leukemia	No	African American	
*BRCA2*	c.1296_1297delGA	60–69	No		No	Caucasian	
*BRCA2*	p.D2723H	30–39	No		No	Caucasian	
*BRCA2*	c.7865dupA	40–49	No	Bladder	No	Asian	
*BRCA2*	c.4456_4459delGTTA	40–49	Yes		No	African American	
*BRCA2*	c.3257_3258delTA	40–49	Yes		No	African American	
*BRCA2*	c.5164_5165delAG	50–59	No		No	African American	
*BRCA2*	c.5722_5723delCT	50–59	No	Tonsil/NOS	No	Caucasian	
*BRCA2*	c.5946delT	50–59	No	Ureter	No	Ashkenazi Jewish	
*BRCA2*	5′UTR_EX15del	50–59	No		No	Ashkenazi Jewish	
*BRCA2*	c.8297delC	50–59	No	Lymphoma	No	Caucasian	
*BRCA2*	c.8297delC	50–59	No	Skin	No	Caucasian	
*BRCA2*	c.8331+1G>A	50–59	No	Pancreas/melanoma	No	Caucasian	
*BRCA2*	p.E1953*	50–59	No		No	Caucasian	
*BRCA2*	c.6938-1G>A	50–59	No	Tonsil	No	Mixed ethnicity	
*BRCA2*	c.5799_5802delCCAA	60–69	No		No	Hispanic	
*BRCA2*	c.5130_5133delTGTA	60–69	No		No	Caucasian	
*BRCA2*	5′UTR_EX1del	60–69	No		No	Caucasian	
*BRCA2*	c.6676_6677delGA	60–69	No		No	Caucasian	
*BRCA2*	c.5722_5723delCT	60–69	No	Prostate	No	Caucasian	
*BRCA2*	c.7977-1G>C	60–69	No		Yes	Caucasian	
*BRCA2*	c.6591_6592delTG	60–69	No		No	Caucasian	
*BRCA2*	c.4940_4941delCA	60–69	No		No	Mixed ethnicity	
*BRCA2*	p.E1308*	60–69	No		No	Unknown	
*BRCA2*	c.1813dupA	60–69	No		No	Caucasian	
*BRCA2*	c.5350_5351delAA	60–69	Yes		No	Caucasian	
*BRCA2*	p.V159M	60–69	No		No	Hispanic	
*BRCA2*	c.9117G>A	60–69	No		No	Caucasian	
*BRCA2*	p.R2336P	60–69	No		No	Other	
*BRCA2*	c.8374_8384del11insAGG	60–69	No	Prostate	No	Caucasian	
*BRCA2*	p.R2520*	70–79	No	Prostate	Yes	Caucasian	
*BRCA2*	p.E3111*	70–79	Yes		Yes	African American	
*BRCA2*	c.4876_4877delAA	70–79	Yes	Colon/lymphoma	No	Caucasian	
*BRCA2*	c.778_779delGA	70–79	No		No	Caucasian	
*BRCA2*	p.R2659K	70–79	No	Prostate	No	Caucasian	
*BRCA2*	c.3975_3978dupTGCT	70–79	No		No	Caucasian	
*BRCA2*	p.Q2859*	70–79	No		No	Caucasian	
*BRCA2*	p.E49*	70–79	No		No	Mixed ethnicity	
*BRCA2*	c.9435_9436delGT	70–79	No	Melanoma/skin	No	Caucasian	
*BRCA2*	c.6068_6072delACCAG	70–79	No	Bladder	No	Caucasian	
*BRCA2*	c.1929delG	70–79	No	Melanoma/skin	No	Caucasian	
*BRCA2*	c.3975_3978dupTGCT	70–79	No	Colon/lung	Yes	Unknown	
*BRCA2*	p.Q3026*	90–99	No		Yes	Mixed ethnicity	
*BRCA2*	p.R2520*	90–99	No		No	Caucasian	
*BRCA2*	c.5946delT	NOS	No		No	Unknown	
*BRCA2*	c.4876_4877delAA	NOS	Yes		No	Hispanic	
*BRCA2/BRIP1*	c.2808_2811delACAA/p.R798*	70–79	No		Yes	Caucasian	
*CHEK2*	c.591delA	60–69	No		NP	Caucasian	
*CHEK2*	p.R117G	40–49	No		No	Caucasian	
*CHEK2*	c.1100delC	40–49	No		No	Caucasian	
*CHEK2*	c.1100delC	40–49	No		No	Caucasian	
*CHEK2*	c.1100delC	40–49	No		No	Caucasian	
*CHEK2*	p.T476M	50–59	No	Melanoma	No	Unknown	
*CHEK2*	c.1100delC	50–59	No	Kidney	No	Caucasian	
*CHEK2*	p.I157T	50–59	No		No	Caucasian	
*CHEK2*	p.T476M	50–59	No	Colon	No	Caucasian	
*CHEK2*	p.I157T	50–59	No		No	Caucasian	
*CHEK2*	c.1100delC	50–59	No		No	Caucasian	
*CHEK2*	p.I157T	50–59	No		No	Ashkenazi Jewish	
*CHEK2*	c.1100delC	60–69	No	Leukemia	No	Caucasian	
*CHEK2*	p.S428F	60–69	No		No	Ashkenazi Jewish	
*CHEK2*	c.1100delC	60–69	No		No	Caucasian	
*CHEK2*	p.Q29*	70–79	No		No	Caucasian	
*CHEK2*	p.S428F	70–79	No		Yes	Ashkenazi Jewish	
*CHEK2*	p.I157T	70–79	No		No	Caucasian	
*CHEK2*	p.I157T	70–79	No		No	Caucasian	
*CHEK2*	c.1100delC	<59	No		Yes	Caucasian	
*CHEK2/PALB2*	p.I157T/c.172_175delTTGT	70–79	No		No	Caucasian	
*MRE11A*	c.1867+2T>C	70–79	No		No	Caucasian	
*NBN*	c.657_661delACAAA	70–79	No		No	Caucasian	
*NF1*	p.R1276*	50–59	No		NP	African American	
*NF1*	p.Q1070*	30–39	No		No	African American	NF1 clinical dx
*NF1*	c.1721+3A>G	40–49	No	Leukemia/pheochromocytoma/lymphoma	No	Mixed ethnicity	Son noted to have NF1 clinical dx
*PALB2*	c.661_662delinsTA	50–59	No	Thyroid	No	Caucasian	
*PALB2*	p.Y1183*	50–59	No		No	Ashkenazi Jewish	
*PALB2*	c.93dupA	60–69	No		No	Caucasian	
*RAD51D*	c.270_271dupTA	80–89	No		No	Asian	

^a^ *NP* not provided

**Table 3:** Frequency of pathogenic variants in breast cancer genes in overall MBC cohort (*n* = 708)

Gene	No prior *BRCA1/2* testing			Prior *BRCA1/2* testing			All MBC		
	Total pathogenic/likely pathogenic variants	Total tested^a^	%	Total pathogenic/likely pathogenic variants	Total tested^a^	%	Total pathogenic/likely pathogenic variants	Total tested^a^	%
*BRCA2*	53	480	11.0	2	197	1.0	55	677	8.1
*CHEK2* (all)	16	386	4.1	6	195	3.1	22	581	3.8
*CHEK2* (excluding I157T)	11	386	2.8	5	195	2.6	16	581	2.8
*CHEK2* (I157T only)	5	386	1.3	1	195	0.5	6	581	1.0
*ATM* ^b^	6	390	1.5	0	196	0.0	6	586	1.0
*BRCA1*	6	480	1.3	0	197	0.0	6	677	0.9
*NF1*	2	354	0.6	1	158	0.6	3	512	0.6
*PALB2*	2	417	0.5	3	204	1.5	5	621	0.8
*RAD51D*	1	354	0.3	0	158	0.0	1	512	0.2
*BRIP1*	1	370	0.3	0	194	0.0	1	564	0.2
*MRE11A*	1	370	0.3	0	194	0.0	1	564	0.2
*NBN*	1	370	0.3	0	194	0.0	1	564	0.2
*BARD1*	0	370	0.0	2	194	1.0	2	564	0.4

^a^The total number of men tested varies by gene, as not all men were tested by the same panel of genes

^b^ *ATM* biallelic individual was counted only once

### Diagnostic yield

To assess the diagnostic yield of MGPT for MBC patients, results were analyzed for patients tested for all 16 breast cancer genes (*n* = 512) (Table 4). The overall mutation-positive rate for breast cancer susceptibility genes for patients with no prior *BRCA1/2* testing reported was 18.1% (*N* = 64/354), with 1.1% (*n* = 4) of patients carrying pathogenic variants in two different breast cancer genes. The overall mutation-positive rate for breast cancer susceptibility genes for patients with prior *BRCA1/2* testing reported was 7.6% (*N* = 12/158), with 1 patient carrying mutations in two different breast cancer genes. Of note, two patients in this group tested positive for *BRCA2* gross deletions that were not previously detected because gross deletion/duplication analysis had not been previously performed.

**Table 4:** Findings among MBC patients tested for 16 breast cancer genes (*n* = 512)

Result category	No prior BRCA testing (*n* = 354)		Prior BRCA testing (*n* = 158)	
	*N*	%	*N*	%
Pathogenic/likely pathogenic variant	64	18.1	12	7.6
Pathogenic/likely pathogenic variant(s) in a single gene	60^a^	16.9	11	7.0
*BRCA1/2*	39	11.0	2	1.3
Non-*BRCA1/2*	21	5.9	9	5.7
Pathogenic/likely pathogenic variant(s) in multiple genes	4	1.1	1	0.6
Combination of *BRCA1/2* and non-*BRCA1/2* genes	3	0.8	0	0.0
Multiple non-*BRCA1/2* genes	1	0.3	1	0.6
Pathogenic/likely pathogenic variant + VUS	16	4.5	5	3.2
VUS only	59	16.7	34	21.5
Negative	231	65.3	112	70.9

^a^59 had a single pathogenic/likely pathogenic variant and 1 had biallelic *ATM* mutations

### Clinical history comparisons

**Fig. 1:**
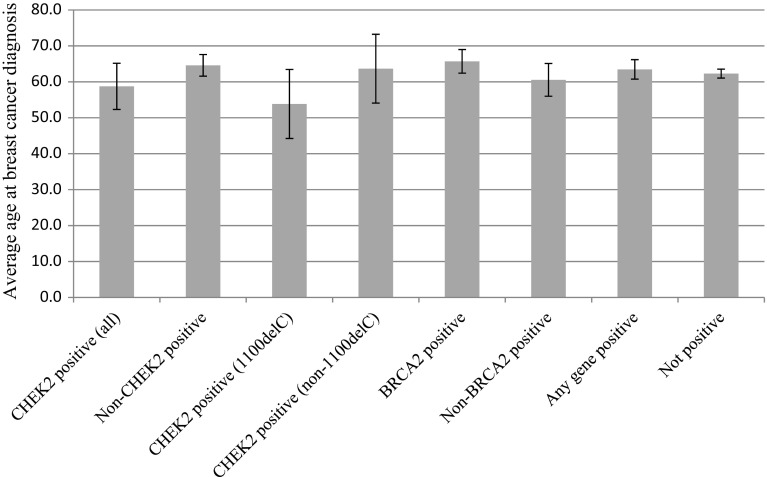
Average age at breast cancer diagnosis based on test result

**Fig. 2:**
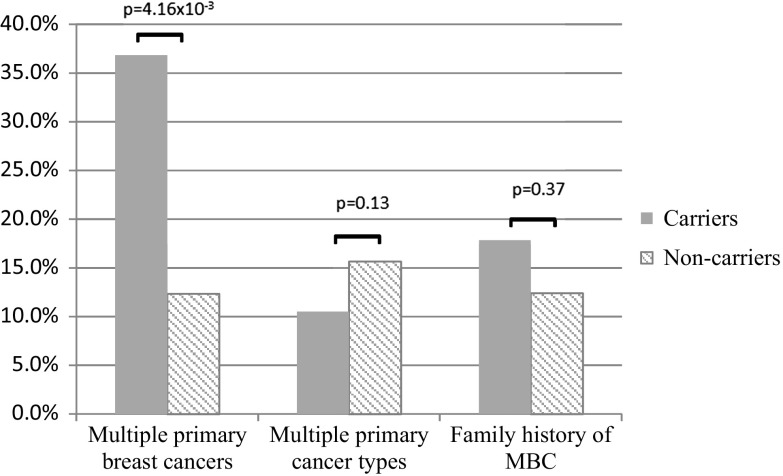
Clinical histories of pathogenic carriers versus non-carriers

The average age of diagnosis was similar for men with (63.5 ± 2.7 years) and without (62.3 ± 1.2 years; *p* = 0.43) pathogenic variants (Fig. 1). In addition, there was no significant difference in history of multiple primary cancers between patients with and without pathogenic variants (*p* = 0.13) (Fig. 2). However, patients with pathogenic variants were more likely to report multiple primary breast cancers (*p* = 4.16 × 10^−3^), with *BRCA2* accounting for all cases. There was no significant difference in family history of MBC (*p* = 0.37).

The average age of diagnosis for men with any *CHEK2* pathogenic variants (58.8 ± 6.4 years) was not significantly different from men with non-*CHEK2* pathogenic variants (64.6 ± 3.0 years; *p* = 0.09) or from men who did not test positive (62.3 ± 1.2 years; *p* = 0.26); however, *CHEK2* 1100delC carriers had a significantly lower average age of diagnosis (53.8 ± 9.6 years) compared to men with non-*CHEK2* variants (*p* = 0.03). No significant differences were observed between average age at breast cancer diagnosis for *CHEK2* 1100delC carriers compared to other *CHEK2* pathogenic variants (63.7 ± 9.6 years; *p* = 0.09) or to men who did not test positive (*p* = 0.07), though these trended toward significance.

### Gene-specific risks of MBC

Case–control analyses were performed based on sequencing results from 421 Caucasian MBC patients and 26,911 ExAC NFE-non TCGA controls. Pathogenic variants in *BRCA2* and *CHEK2* were significantly associated with increased risk of MBC (*BRCA2* OR = 13.9, *p* = 1.92 × 10^−16^; *CHEK2* OR = 2.43, *p* = 1.82 × 10^−3^) (Table 5). Additional studies evaluating risks associated with *CHEK2* 1100delC and excluding common/low-risk missense variants (p.Ile157Thr and p.Ser428Phe) showed that truncating variants in *CHEK2* are associated with moderately increased risks of MBC (OR = 3.8; 95% CI 2.1–6.8; *p* = 1.51 × 10^−4^) (Table 5). Variants in *PALB2* were also significantly associated with a high risk of MBC (OR = 6.6, *p* = 0.013) (Table 5). However, this risk estimate is uncertain due to small numbers of MBCs with pathogenic variants (95% CI 1.70–21.09). Interestingly, few pathogenic variants were identified in *ATM* and *BRCA1*, which are commonly mutated in female familial breast cancer. No significant associations with MBC risks were observed. Sensitivity analyses excluding MBCs with prior testing of *BRCA1/2* showed very similar effects for pathogenic variants in these genes (Online Resource 3).

**Table 5:** Breast cancer risks associated with pathogenic variants pooled by gene among Caucasian male breast cancer cases

Gene	Ambry cases		ExAC controls		Cancer risk			
	Mutated alleles	Cases	Mutated alleles	Cases	OR	95% CI lower	95% CI upper	*p* value
*ATM*	2	421	90	26,644	1.4	0.3	5.1	0.66
*BRCA1*	2	394	74	26,911	1.8	0.3	6.8	0.30
*BRCA2*	21	394	105	26,791	13.9	8.5	22.5	1.92 × 10^−16^
*CHEK2* All	17	421	424	25,215	2.4	1.4	3.9	1.82 × 10^−3^
*CHEK2*_c.1100delC	8	421	127	25,215	3.8	1.7	7.8	1.82 × 10^−3^
*CHEK2* W/O I157T/S428F	10	421	163	25,215	3.7	1.9	7.0	6.24 × 10^−4^
*CHEK2* W/O I157T	12	421	191	25,215	3.8	2.1	6.8	1.51 × 10^−4^
*CHEK2* I157T	5	421	233	25,215	1.3	0.5	3.0	0.60
*PALB2*	3	421	29	26,869	6.6	1.7	21.1	0.013

## Discussion

Previously reported cohorts of MBC patients undergoing MGPT have included 22–51 cases [8, 9], making this the largest reported collection to date of MBC patients undergoing MGPT. As expected, *BRCA2* accounted for the largest percentage of pathogenic variants, whereas the observed frequency of *CHEK2* pathogenic variants (4.1%) was greater than expected based on previous reports of MBC in *CHEK2* cohorts. These findings support recent reports of the *CHEK2* pathogenic variant frequencies among MBC cases in the MGPT setting (4.5–7.8%) [8, 9]. While *BRCA2* is an established MBC susceptibility gene, literature regarding an association of *CHEK2* with MBC is conflicting. Despite an initial report in 2002 concluding that *CHEK2* 1100delC is associated with a tenfold risk for MBC [13], and a subsequent report of an association between 1100delC and MBC in the Dutch population [14], multiple other studies have not affirmed this association [15–22]. The limited number of probands affected with MBC (i.e., under 100 in most studies) and the lack of full sequencing of *CHEK2* in published cohorts may explain these conflicting reports. In the current study, *CHEK2* protein-truncating variants were associated with a 3.8-fold increased risk for MBC, which is highly consistent with findings from the studies of breast cancer families. Confidence intervals ranged from 2.1 to 6.8 suggesting that *CHEK2* is a moderate risk gene for MBC. In contrast, *BRCA2* pathogenic variants were associated with much higher risks of MBC (OR = 13.9; 95% CI 8.5–22.5).

Multiple *ATM* and *PALB2* pathogenic variants were also detected among MBC patients in this cohort. To our knowledge, this is only the second report of MBC in *ATM* heterozygotes [8] and the first report of MBC in a patient with ataxia-telangiectasia. Of note, two of the five *ATM* pathogenic variant carriers in the refined 16-gene subgroup were multiple pathogenic variant carriers, including one *ATM* biallelic carrier and one *ATM/BRCA2* carrier. In the larger cohort, there was also one additional *ATM/BRCA2* carrier. Furthermore, *ATM* pathogenic variants were not significantly associated with MBC (Table 5). These observations suggest *ATM* may act as an MBC risk modifier. There are multiple previous reports of *PALB2* pathogenic variants among MBC families, with a frequency ranging from 0.8 to 6.4%, although most reports have not met statistical significance [23, 34–37]. One study reported that *PALB2* pathogenic variant carriers were four times more likely than *PALB2*-negative patients to have a relative with MBC (*p* < 0.001) [34]. In addition, Antoniou et al. reported an eightfold increased risk for MBC in *PALB2* carriers from moderate- and high-risk families; however, this did not reach statistical significance (*p* = 0.08) [35]. Consistent with both reports, *PALB2* pathogenic variants in the current study were associated with a 6.6-fold increased risk of MBC (Table 5). Further association studies, in families and in the general population, are needed to confirm the association of genes such as *ATM* and *PALB2* with MBC and to calculate more precise breast cancer risks for males with pathogenic variants in these genes.

Five (1.41%) of the MBC patients in the refined subgroup with no prior *BRCA* testing carried multiple pathogenic variants. As mentioned above, one of the MBC patients had biallelic *ATM* pathogenic variants and was noted to have a clinical history of ataxia-telangiectasia on the requisition form. Three of the multiple pathogenic variant carriers had a combination of pathogenic variants in one high-risk gene and one moderate risk gene: *BRCA1/CHEK2*, *BRCA2/ATM*, and *BRCA2/BRIP1*. The other multiple pathogenic variant carriers had mutations in two moderate-risk genes: *CHEK2/PALB2*. Excluding skin cancer, only the *BRCA2/ATM* pathogenic variant carrier reported multiple primary cancers (MBC and prostate cancer). The percentage of multiple pathogenic variants in this cohort and other reported MBC cohorts appears to be similar to multiple pathogenic variants in female breast cancer cohorts [8, 9].

Due to the relatively low number of pathogenic variants in other non-breast cancer genes in this cohort, it is difficult to assess whether MBC is an unrecognized component of the cancer spectra for these genes. Interestingly, several men tested positive for a pathogenic variant in genes associated with a syndromic presentation, including *APC* and *SDHA.* These patients did not have classical presentation of the associated syndromic features, indicating that gene-specific testing likely would not have been considered (Online Resource 2). Breast cancer—male or female—is not currently considered a component of the cancer spectra for these genes. While identification of a pathogenic variant in these cases is likely to impact medical management for other cancers, the result offers little insight into the most appropriate management of MBC risk, specifically, or whether other males in the family should be considered for testing and/or high-risk breast cancer screening.

No *PTEN* pathogenic variants were detected among MBC probands, despite previous reports of *PTEN* carriers with MBC. Since *PTEN* pathogenic variants are typically associated with Cowden syndrome (i.e., the presence of macrocephaly and characteristic mucocutaneous features in addition to cancer predisposition), it is likely that MBC patients with clinical histories suggestive of Cowden syndrome would be referred for *PTEN* testing alone rather than MGPT. Therefore, the absence of *PTEN* mutations in this cohort does not necessarily contradict previous reports. Similarly, pathogenic variants were not identified in *TP53* or *CDH1* in this cohort. While male breast cancer is not a major feature associated with either of these genes, it is possible that men with clinical histories suggestive of Li–Fraumeni syndrome or Hereditary Diffuse Gastric Cancer syndrome would have had single gene testing instead of MGPT, potentially introducing ascertainment bias with respect to these genes.

With the exception of men with *CHEK2* 1100delC, age of diagnosis was not predictive of positive test results. Although the number of men carrying the *CHEK2* 1100delC in this cohort is small, the significantly younger age of diagnosis in this subset may indicate that men with this specific pathogenic variant may warrant surveillance and/or a higher index of suspicion for male breast cancer at a younger age compared to men with other pathogenic variants. Family history of MBC and additional primary cancer diagnoses were also not predictive of positive results in this cohort, consistent with current NCCN *BRCA1/2* testing guidelines which recommend testing for MBC patients regardless of age at diagnosis or other clinical history. In contrast, multiple breast primary cancers were only identified in *BRCA2* pathogenic variant carriers in this cohort, suggesting a role for first-line *BRCA1/BRCA2* testing in men with this presentation.

The identification of pathogenic variants in MBC patients may have clinical implications both for the affected men and their relatives. For example, breast cancer screening is recommended for *BRCA1/BRCA2*-positive men, beginning at age 35, and increased colon surveillance is recommended for *CHEK2*-positive individuals [1]. Several of the pathogenic variants identified in this cohort are associated with risks for other cancers, and their identification allows for increased surveillance which may lead to earlier detection of subsequent cancers. Many of the pathogenic variants identified in this cohort also carry significant risks for breast and ovarian cancer in women. Therefore, identification of variants in MBC patients allows for testing at-risk family members and increased surveillance and/or risk-reducing surgeries for positive relatives. Given the clinical implications for patients and their families, there appears to be utility in choosing a MGPT approach for MBC patients.

There are several limitations to this study. While previous *BRCA1/2* testing was controlled for in this analysis, it is possible that previous *BRCA1/2* testing was underreported in this group. Clinical history was ascertained by information reported on test requisition forms, and were verified by pedigree review when provided. As such, the analysis of secondary cancers and family history of cancer may be limited by the accuracy and completeness of the data provided. However, results from a recent study demonstrated that clinical history on test requisition forms at Ambry Genetics is highly accurate and complete for probands and highly accurate for relatives, with completeness correlating with relationship to the proband (i.e., more complete for first- and second-degree relatives and less complete for third-degree relatives and beyond) [38]. In addition, as this is a retrospective review of men selected for different clinical genetic tests and may over-represent male breast cancer cases in the setting of a family history also indicative of a hereditary predisposition for cancer, the results of the study may be influenced by ascertainment bias or be specifically applicable to a high-risk population. Finally, segregation data in families with multiple cases of male breast cancer and in families with multiple pathogenic variants from this cohort are not available. Segregation data could potentially clarify the association between male breast cancer and the identified pathogenic variants in these families.

Results from this study build upon the current understanding of hereditary susceptibility to MBC. These data lend support to a MGPT approach for MBC patients regardless of age at diagnosis, history of multiple primary cancers, and family history of MBC. Furthermore, these data support *CHEK2* as a MBC susceptibility gene. The observed pathogenic variant frequency in this MBC cohort highlights the immediate need for studies investigating the most appropriate screening and risk management tools for MBC patients, particularly in cases with pathogenic variants in genes beyond *BRCA1/2*.

## Electronic supplementary material

Below is the link to the electronic supplementary material.

Supplementary material 1 (DOCX 31 kb)
