# Supplementary material for: Male breast cancer in a multi-gene panel testing cohort: insights and unexpected results
Source: Breast Cancer Res Treat. 2016 Dec 22;161(3):575–86. doi: 10.1007/s10549-016-4085-4 (PMC5241330; doi:10.1007/s10549-016-4085-4)
Supplement: Supplementary file 1 — Supplementary material 1 (DOCX 31 kb) [file 10549_2016_4085_MOESM1_ESM.docx]

**Supplementary Materials**

Supplemental Table 1. Genes included on multigene panel tests

| **Multi-Gene Panel Test** | **Genes Included** |
| --- | --- |
| BRCAplus | *BRCA1, BRCA2, CDH1, PALB2^a^, PTEN, STK11^b^, TP53* |
| BRCAplus Expanded | *ATM, BRCA1, BRCA2, CDH1, CHEK2, PALB2,PTEN, TP53* |
| BreastNext | *ATM, BARD1, BRCA1^c^, BRCA2^c^, BRIP1, CDH1, CHEK2, MRE11A, MUTYH, NBN, NF1^d^, PALB2, PTEN, RAD50, RAD51C, RAD51D^d^, STK11^b^, TP53* |
| GYNPlus | *BRCA1, BRCA2, BRIP1^g^, EPCAM^e^, MLH1, MSH2, MSH6, PALB2^g^, PMS2, PTEN, RAD51C^g^, RAD51D,^g^ TP53* |
| OvaNext | *ATM, BARD1, BRCA1^c^, BRCA2^c^, BRIP1, CDH1, CHEK2, EPCAM^e^, MLH1, MRE11A, MSH2, MSH6, MUTYH, NBN, NF1^d^, PALB2, PMS2, PTEN, RAD50, RAD51C, RAD51D^d^, SMARCA4^f^, STK11, TP53* |
| PancNext | *APC, ATM, BRCA1, BRCA2, CDKN2A, EPCAM^e^, MLH1, MSH2, MSH6, PALB2, PMS2, STK11, TP53* |
| CancerNext | *APC, ATM, BARD1, BRCA1^c^, BRCA2^c^, BRIP1, BMPR1A, CDH1, CDK4^d^, CDKN2A^d^, CHEK2, EPCAM^e^, GREM1^e,f^, MLH1, MRE11A, MSH2, MSH6, MUTYH, NBN, NF1^d^, PALB2, PMS2, POLD1^f^, POLE^f^, PTEN, RAD50, RAD51C, RAD51D^d^, SMAD4, SMARCA4^f^, STK11, TP53* |
| CancerNext-Expanded | *APC, ATM, BAP1^f^, BARD1, BRCA1, BRCA2, BRIP1, BMPR1A, CDH1, CDK4, CDKN2A, CHEK2, EPCAM^e^, FH, FLCN, GREM1^e,f^, MAX, MEN1, MET, MITF^h^, MLH1, MRE11A, MSH2, MSH6, MUTYH, NBN, NF1, PALB2, PMS2, POLD1^f^, POLE^f^, PTEN, RAD50, RAD51C, RAD51D, RET, SDHA, SDHAF2, SDHB, SDHC, SDHD, SMAD4, SMARCA4^f^, STK11, TMEM127, TP53, TSC1, TSC2, VHL* |

^a^*PALB2* included for panels ordered on or after 10/1/2015

^b^*STK11* removed for panels orders authorized on or after 8/1/14
^c^*BRCA1* and *BRCA2* included for panels ordered on or after 6/13/13
^d^*NF1, RAD51D, CDKN2A,* and *CDK4* included for panels ordered on or after 10/18/13

^e^*EPCAM* and *GREM1* include reporting of selected gross deletions/duplications only

^f^*BAP1, GREM1, POLD1, POLE*, and *SMARCA4* included for panels ordered on or after 5/18/15

*^g^BRIP1*, *PALB2*, *RAD51C*, and *RAD51D* included for panels ordered on or after 6/1/16

^h^For MITF only the status of the c.952G>A (p.E318K) alteration is analyzed and reported

Online Resource 2. Findings in genes not currently associated with breast cancer

| **Positive Gene(s)** | **Pathogenic Variant(s)** | **First Breast Cancer Age** | **Bilateral/ Multiple Breast Cancers** | **Other Cancer(s)** | **Family History of MBC** | **Ethnicity** | **Notes** |
| --- | --- | --- | --- | --- | --- | --- | --- |
| *APC* | c.6281delC | 20-29 | no | None | no | Caucasian | 2-5 adenomatous polyps at age 62 |
| *APC* | p.I1307K | 50-59 | no | None | no | Ashkenazi Jewish |  |
| *APC* | p.I1307K | 60-69 | no | None | yes | Caucasian |  |
| *APC* | p.I1307K | 70-79 | no | Esophageal/Bladder | no | Ashkenazi Jewish |  |
| *MITF* | p.E318K | 60-69 | no | None | no | Caucasian |  |
| *SDHA* | p.R31* | 50-59 | no | Colorectal/Kidney | No | Caucasian |  |

Online Resource 3. Breast cancer risks associated with pathogenic variants pooled by gene among Caucasian male breast cancer cases

|  | Ambry cases | | ExAC controls | |  | Cancer Risk | | |
| --- | --- | --- | --- | --- | --- | --- | --- | --- |
| Gene | Mutated Alleles | Cases | Mutated Alleles | Cases | OR | 95%CI lower | 95%CI upper | p-value |
| *ATM* | 2 | 268 | 90 | 26644 | 2.2 | 0.39 | 8.1 | 0.23 |
| *BRCA1* | 2 | 263 | 74 | 26911 | 2.8 | 0.48 | 10.3 | 0.17 |
| *BRCA2* | 21 | 263 | 105 | 26791 | 21.19 | 12.9 | 34.3 | 6.21x10^-20^ |
| *CHEK2* All | 12 | 268 | 424 | 25215 | 2.7 | 1.49 | 4.9 | 2.50x10^-3^ |
| *CHEK2*_c.1100delC | 5 | 268 | 127 | 25215 | 3.7 | 1.44 | 9.0 | 0.01 |
| *CHEK2* W/O I157T/S428F | 7 | 268 | 163 | 25215 | 4.1 | 1.88 | 8.6 | 2.30x10^-3^ |
| *CHEK2* I157T | 4 | 268 | 233 | 25215 | 1.6 | 0.55 | 4.3 | 0.32 |
| *PALB2* | 1 | 268 | 29 | 26869 | 3.5 | 0.17 | 19.7 | 0.26 |

All cases previously tested for *BRCA1* and *BRCA2* were excluded.

**Supplementary Methods**

Family history

Family history was limited to first and second-degree relatives. Families with breast or ovarian cancer in two or more individuals, on the same parental side, were considered positive for family history for each cancer. *MUTYH* mutations were excluded from the analysis because *MUTYH*-associated polyposis is considered an autosomal recessive condition.

Data cleaning and filtering rules

**Breast cancer cases**

- Restricted to single individual per family
- Restricted to breast cancer
- Restricted to male
- Included loss of functional variants ((Nonsense, frameshift, +/-1,2 splice) with minor allele frequency (MAF<0.003). Included the known common pathogenic variant CHEK2 1100delC.
- Included missense variants classified as pathogenic in ClinVar database by two or more clinical genetics group (Ambry, SCRP, InVitae, GeneDX, Emory and InSiGHT). Classifications submitted by OMIM, BIC, or other non-clinical groups were not considered in classification criteria.
- Excluded VLP
- Excluded mosaic mutations from allele count
- Excluded multiple mutation carriers (except for carriers with a MUTYH variant). Not scored in ExAC.
- Excluded individuals with structural variants classified as “pathogenic” (such as gene deletions, exon/intron deletion mutations). Not validated in ExAC.
- Excluded individual carriers of PMS2 Mutations located in exons 9, 11-15 from both Ambry Genetics cases and ExAC controls because these exons are shared by PMS2 pseudogene.
- Excluded CHEK2 homozygotes.
- Excluded ExAC non-PASS recurrent variants (tested in >20,000 ExAC alleles, allele count in ExAC>8)
- Excluded variants with low penetrance: APC (p.I1307K); BRCA1 (p.R1699Q); CHEK2 (p.I157T, p.S428F); PMS2 (c.736_741del6ins11); PTEN (p.P354Q) TP53 (p.R283H, 5'UTR_EX1del, p.R181H, p.R156H).
- Removed truncating variants not influenced by nonsense mediated RNA decay (NMD) - +55 of penultimate exon and all of last exon, if no functional domain.
- Restricted to Caucasian and Ashkenazi Jewish

**ExAC variant cleaning**

Exclusions

- Restricted to ExAC NFE-non TCGA data
- Excluded specific ExAC PASS/non-PASS variants classified as pathogenic
  1. Exclude ExAC non-PASS variants classified as pathogenic and with multiple repetitive sequences. Example: MSH2_c.942+2_942+6del5, MSH2_c.942+2_942+4delTAA, MSH2_c.942+2_942+5delTAAA, MSH2_c.942+2_942+3delTA, MSH2_c.942+2_942+8del7 MSH2_c.942+2_942+7del6.
  2. Exclude recurrent (AC>8) ExAC non-PASS pathogenic variants observed in <20,000 alleles.

Variant classification

- Classified as pathogenic “mutations”
  - Include loss-of-function variants (Nonsense, frameshift, +/-1,2 splice) with minor allele frequency (MAF<0.003).
  - Include known common pathogenic variant CHEK2 1100delC (MAF>0.003)
  - Variants classified as VLB (variant likely benign) by Ambry Genetics or benign or neutral by ClinVar were excluded from ExAC
  - Missense variants classified as pathogenic in ClinVar database by two or more clinical genetics group (Ambry, SCRP, InVitae, GeneDX, Emory and InSiGHT) were classified as pathogenic in ExAC. Classifications submitted by OMIM, BIC, or other non-clinical groups.
